# Electromagnetic radiation therapy for Parkinson’s disease tremor reduction- systematic reviews and Bayesian meta-analyses for comparing the effectiveness of electric, magnetic and light stimulation methods

**DOI:** 10.1186/s12984-023-01255-z

**Published:** 2023-09-26

**Authors:** Seyedeh Marzieh Hosseini, Sajjad Farashi, Saeid Bashirian

**Affiliations:** 1 Autism Spectrum Disorders Research Center, Hamadan University of Medical Sciences, Hamadan, Iran; 2 Neurophysiology Research Centre, Hamadan University of Medical Sciences, Hamadan, Iran; 3 Social Determinants of Health Research Center, Hamadan University of Medical Sciences, Hamadan, Iran

**Keywords:** Systematic review, Parkinson’s disease, Tremor reduction, Electric stimulation, Magnetic stimulation, Light therapy

## Abstract

**Purpose:**

Tremor is one of the key characteristics of Parkinson’s disease (PD), leading to physical disabilities and often showing limited responses to pharmacological treatments. To suppress tremors in PD patients, several types of non-invasive and non-pharmacological methods have been proposed so far. In the current systematic review, three electromagnetic-based radiation strategies including electrical stimulation, magnetic stimulation, and light stimulation methods were reviewed and compared.

**Methods:**

Major databases were searched to retrieve eligible studies. For the meta-analysis, a random-effect Bayesian framework was used. Also, heterogeneity between studies was assessed using I^2^ statistic, prediction interval, and tau2. Publication bias was assessed using funnel plot, and the effectiveness of methods for reducing tremor was compared using network Bayesian meta-analysis.

**Results and conclusion:**

Thirty-one studies were found for qualitative analysis, and 16 studies were found for quantitative synthesis. Based on the suppression ratio, methods can be ordered as electrical stimulation, light therapy, and magnetic stimulation. Furthermore, the results showed that electrical and magnetic stimulation were more effective for tremor suppression at early stages of PD, while light therapy was found to be more effective during the later stages of PD.

**Supplementary Information:**

The online version contains supplementary material available at 10.1186/s12984-023-01255-z.

## Introduction

In Parkinson’s disease (PD), the dopaminergic neurons that produce dopamine are damaged. Dopamine is required for the smooth control of muscle movements. In this regard, due to dopamine insufficiency, PD patients may experience symptoms such as tremors, muscle rigidity, slowness of movements [1], and balance problems. Also, other functions such as eye movement may be affected during PD [2].

Tremors in PD patients are characterized by involuntary, rhythmic, and roughly sinusoidal oscillations and are responsible for many functional disabilities [3–5]. The well-known treatments for reducing tremor include drug therapy, surgery, deep brain stimulation (DBS), and thalamic deep brain stimulation [6, 7]. Despite their advantages, drug-based treatments are not completely effective and can cause several types of side effects. Furthermore, other methods are invasive and post-operative, where bleeding may occur. In recent decades, several researchers have been motivated by non-drug and non-surgical methods for attenuating tremors [6].

Disrupting tremor signals by applying electromagnetic waves to nerves or muscles is a potential non-pharmacological and non-invasive method for tremor reduction in PD patients. Functional electrical stimulation [8–10], light therapy [11], and transcranial magnetic stimulation [12] are among such interventions. Applying lights with different frequencies and intensities has been proposed for reducing PD tremors [13–15]; however, there are inconsistencies between studies on the effectiveness of light therapy [13, 16]. Several studies were also focused on electrical or magnetic stimulation of nerves or muscles in a non-invasive manner (for review see [17] and [18]).

In this systematic review, the current knowledge on three types of electromagnetic interventions (i.e. electric stimulation, magnetic stimulation, and light stimulation) for tremor reduction in PD patients was updated. Furthermore, using a network meta-analysis framework, the performance of such methods for tremor reduction was compared. It should be noted that this study is part of a comprehensive study related to the non-invasive and non-pharmacological methodologies for tremor reduction in PD patients. Other methodologies such as orthosis, physical therapy, cooling and warming, vibration therapy, and limb weight therapy, which are mainly based on mechanical roles will be published elsewhere.

## Materials and methods

This systematic review was conducted following the Preferred Reporting Items for Systematic Reviews and Meta-analyses (PRISMA) guidelines to identify relevant research papers.

### Search strategy

An initial search was conducted through major databases, including PubMed, Web of Science, Scopus, and Google Scholar search engine, until January 2023. The search terms used were: (Parkinson’s disease OR PD OR Parkinsonism OR Parkinsonian syndrome) AND (tremor OR resting tremor OR postural tremor) AND (reduction OR suppression OR treatment OR inhibition) AND (functional electrical stimulation OR FES OR electrical stimulation OR transcranial magnetic stimulation OR TMS OR electromyography OR EMG OR nerve stimulation OR muscle stimulation) AND (light therapy OR near infrared light OR low level laser OR photobiomodulation OR bright light therapy OR BLT). There are no limitations on article type, language, or publication date.

### Inclusion and exclusion criteria

The studies that satisfied the following inclusion criteria were included in this paper: [1] original research articles, clinical trials, randomized controlled trials, case studies, comparative studies, and pilot studies [2], studies involving PD patients affected by tremor [3], studies in which pre-treatment and post-treatment results for PD subjects were compared or PD individuals were compared with a control group with a correct methodological design and sufficient statistical analysis, and [4] studies in which electric, magnetic or light electromagnetic interventions were used for tremor reduction or inhibition. It should be noted that electromagnetic radiation consists of a spectrum of different waves with varying frequency ranges, including radio waves, microwaves, infrared, visible light, ultraviolet, X-ray, and gamma rays. In this regard, light stimulation can be considered an intervention with an electromagnetic basis. Also, the exclusion criteria were as follows: [1] Review papers, other systematic reviews, and meta-analyses papers related to PD were excluded; however, the reference lists were screened for potential missing eligible studies [2], studies with a small sample size (n < 2) were excluded to avoid insignificant outcomes [3], studies focused on tremor due to reasons other than PD [4], studies involving non-human samples [5], studies that included PD subjects with dementia and [6] studies focused on invasive deep brain stimulation. In this meta-analysis, we excluded case report studies (sample size = 1 or n < 2) from both quantitative and qualitative analyses, since case reports are usually non-blinded and their design lacks randomization, they may be a source of bias [19] and in this way perturb the outcome of the study. Most of the included studies excluded PD samples with dementia, furthermore, dementia has profound effects on brain structures and functions [20]. In this regard, reports for PD cases with dementia were excluded to have at first more homogenous samples and also let future studies check our hypothesis with neuroimaging techniques which are susceptible to the brain structures and functions.

### Study selection

The literature search, title and abstract screening were conducted by two independent authors (SMH and SF) and all the results were collected in EndNote X9. First, the title and abstract of all studies were screened based on the PICO model (Participants: PD patients, Intervention: tremor-suppressing, Comparison: tremor level of PD group, and Outcome: tremor change after intervention). When a paper was published in two languages, we relied on the English version.

The full text of the eligible studies was screened supplemented by a backward search in their reference lists to include any missing studies. Resolving any potential disagreements was achieved through discussion.

### Data extraction

Information from the included studies was extracted by two authors. This information was the first author’s name, publication year, study type, sample size, intervention method, tremor measurement method, outcomes of the study, tremor suppression ratio, size of the effect, 95% confidence interval (CI) and participant information including age, gender, duration of disease, and PD severity. The quality of each study was assessed using quality assessment tools, including the JADAD score [21], NIH quality assessment [22], and the Newcastle-Ottawa Statement scale (NOS) [23].

### Statistical analysis, between-study heterogeneity, and publication bias

Since the number of retrieved studies was relatively small, the Bayesian meta-analysis approach using the Markov chain Monte Carlo (MCMC) sampling procedure was selected for obtaining the pooled effect. The Bayesian framework outperforms frequentist meta-analysis in cases where the number of selected studies is small [24]. For the pooled effect size, the credible interval (CrI), an interval in which the parameter value may fall with a particular probability (95%), was also reported. Weakly informative priors for the effect size and between-study variance were chosen in our hierarchical Bayesian model according to a normal distribution for the effect size and a Half-Cauchy distribution for between-study variance. The effect size (d) for each study was calculated according to the standardized mean difference (SMD) and Cohen’s d. To address the upward bias of Cohen’s d for small samples (which was the case for included studies in our meta-analysis), the corrected Cohen’s d was used [25] as follows.

1 $${d}^{*}=\frac{{M}_{1}-{M}_{2}}{{SD}_{pooled}}\left(\frac{N-3}{N-2.25}\right)\sqrt{\frac{N-2}{N}}$$

In [1], *M*_*i*_ was the mean tremor index for the *i*-th group, *N* was the number of studies, and *SD*_*pooled*_ was the pooled standard deviation. For within-group analysis (assessing the tremor reduction in a single PD group after intervention), groups 1 and 2 were considered as post-intervention and pre-intervention, respectively. Therefore, the negative sign in reported results indicated tremor reduction following the intervention.

The initial iteration for fitting the Bayesian model was 3000. Since the convergence of the Bayesian model is a critical issue, it was checked before any further analysis. In this regard, the posterior predictive check and checking the $\widehat{R}$ values of the estimated parameters were conducted ($\widehat{R}$ should be smaller than 1.05) [24]. In the case of rejecting Bayesian model convergence, larger iterations will be used for fitting the model.

For between-group analysis (comparing tremor reduction between PD and healthy groups), group 1 referred to the PD group. For assessing between-study heterogeneity, different types of measures including Cochran’s Q test, Higgins and Thompson’s I^2^ and H^2^ statistics [26], prediction intervals (PI) [27], and heterogeneity variance (${\tau }^{2}$) [28] were used. When the I^2^ value is smaller than 25%, H^2^ ≤ 1, PI range and ${\tau }^{2}$ does not include zero, there were no symptoms of between-study heterogeneity [24]. In cases of heterogeneity between studies, tests for revealing outlier or influential studies (using *find.outliers* and *InfluenceAnalysis* functions available in R *dmetar package*) as well as subgroup analysis were conducted. Publication bias was assessed using funnel plot, as well as Egger’s regression [29], Begg’s correlation [30], and Thompson’s [31] tests. All analyses were performed in R (version 4.1.2), specifically using *brms, brmstools*, *metaphor, dmetar* and *tidybayes* packages. The significance level of 0.05 and 95% confidence or credible interval were used for reporting statistical analyses.

Since three intervention methods were compared in the current study, a network Bayesian meta-analysis approach was conducted using the *gemtc* R package, JAGS software, and Gibbs sampling procedure. The random effect Bayesian model, four Markov chains for estimating the posterior distribution of parameters and the number of simulation iterations = 100,000 were used. The convergence of the network was assessed using trace plots, posterior effect size estimates and the *Potential Scale Reduction Factor* (PSRF) with the Gelman-Rubin plot. The posterior estimate of effect size should resemble the bell shape of a normal distribution, and the PRSF should converge to zero as the iteration number increases.

**Table 1:** Summary of included studies for non-invasive, non-pharmacological, electromagnetic-based PD tremor reduction techniques (Pre-Post: Pre-Post intervention; HY: Hoehn & Yahr scale; RCT: Randomized control trial; CC: Case-Control; CT: Clinical trials; CD: Cannot be determined, NR: Not reported)

Category	Study (First author, Year)	Study type	Sample size	Corrected Cohen’s d (95%CI)	Tremor Suppression (%)
Light therapy	Hong, 2021	Pre-post	18	UPDRS III: -0.65 (-1.32, 0.02)	28.3 (according to UPDRS III, tremor subscore)
	Hamilton, 2019	Case report	6	CD	50 (number of patients reported improvement)
	Hamilton, 2018	Case report	3	CD	50 (number of patients reported improvement)
	Willis, 2018	CT, Pre-Post	30	UPDRS III: -0.27 (-1.411, 0.863) (Red light); -1.148(-2.368, 0.074) (Polychromatic light)	NR
	Willis, 2007	CT	12	CD	NR
	Paus, 2007	RCT	36	UPDRS III: -0.28(-0.937, 0.337)	NR
Magnetic stimulation	Spagnolo, 2021	RCT	59	UPDRS III: -1.00 (-1.48, -0.54)	22.9 (tremor score); 27.1 (UPDRS III)
	Shi, 2020	Pre-Post	30	CD	NR
	Khedr, 2019	Pre-Post	52	UPDRS III: -0.361(-0.91,0.187) for 1 Hz stimulation; -0.701 (-1.261, -0.141) for 20 Hz stimulation	NR
	Malling, 2019	RCT	36	Tremor intensity index: -2.61 (-0.83, 0.31)	22 (tremor intensity)
	Lu, 2015	CC	10	CD	46.7
	Filipović, 2010	Pre-Post	10	UPDRS III (tremor subscore): -0.03 (-0.91,0.85), UPDRS III: -0.179(-1.06, 0.7),	NR
	Anninos, 2007	Pre-Post	30	HY: -1.135 (-1.698, -0.606)	60
	Britton, 1993	CC	10	CD	NR
Electrical stimulation	Zhang 2023	RCT	13	CD	NR
	Phokaewvarangkul, 2021	Pre-Post	20	UPDRS III(tremor subscore): -0.558(-1.19,-0.074)	NR
	Arruda, 2021	Pre-Post	10	CD	36
	Hao, 2018	Pre-Post	14	CD	71
	Dideriksen, 2017	Pre-Post	5	CD	60
	Hao, 2017	Pre-Post	8	CD	47.97
	Jitkritsadakul, 2017	Pre-Post	15	UPDRS III (tremor): -0.509 (-1.537, 0.52)	NR
	Xu, 2016	Pre-Post	2	Amplitude of joint angles: -0.385 (-3.152, 2.436)	NR
	Jitkritsadakul, 2015	Pre-Post	34	UPDRS III tremor subscore: -0.88 (-1.584, -0.176); Tremor Peak amplitude: -0.558 (-1.243, 0.128); Tremor frequency: -0.206 (-0.88,0.468)	49.57
	Dosen, 2015	Pre-Post	4	CD	71 for motor stimulation; 56.75 for sensory stimulation
	Gallego, 2013	Pre-Post	2	CD	42.56 for tremor amplitude
	Hao, 2013	Pre-Post	10	EMG amplitude: -2.56 (-4.215, -0.879)	63.6
	Maneski, 2011	Pre-Post	4	CD	64.75
	Saavedra-Escalona, 2005	Pre-Post	23	UPDRS III: -0.199 (-0.806, 0.407)	78.26
	Spiegel, 2002	Pre-Post	8	Tremor frequency: Stimulation of opponens pollicis muscle: 0.273 (-1.12,1.66); forearm muscles: 0.386 (-1.13,1.78) Upper arm muscles: 0.356 (-1.04,1.75)	NR
	Gillard, 1999	Pre-Post	3	CD	84.5
	Javidan, 1992	Pre-Post	4	Tremor frequency: 0.043 (-1.92,2.0)	61.5
	Bathien, 1980	Pre-Post	10	CD	NR
	Mones, 1969	Pre-Post	5	CD	NR

## Results

**Fig. 1:**
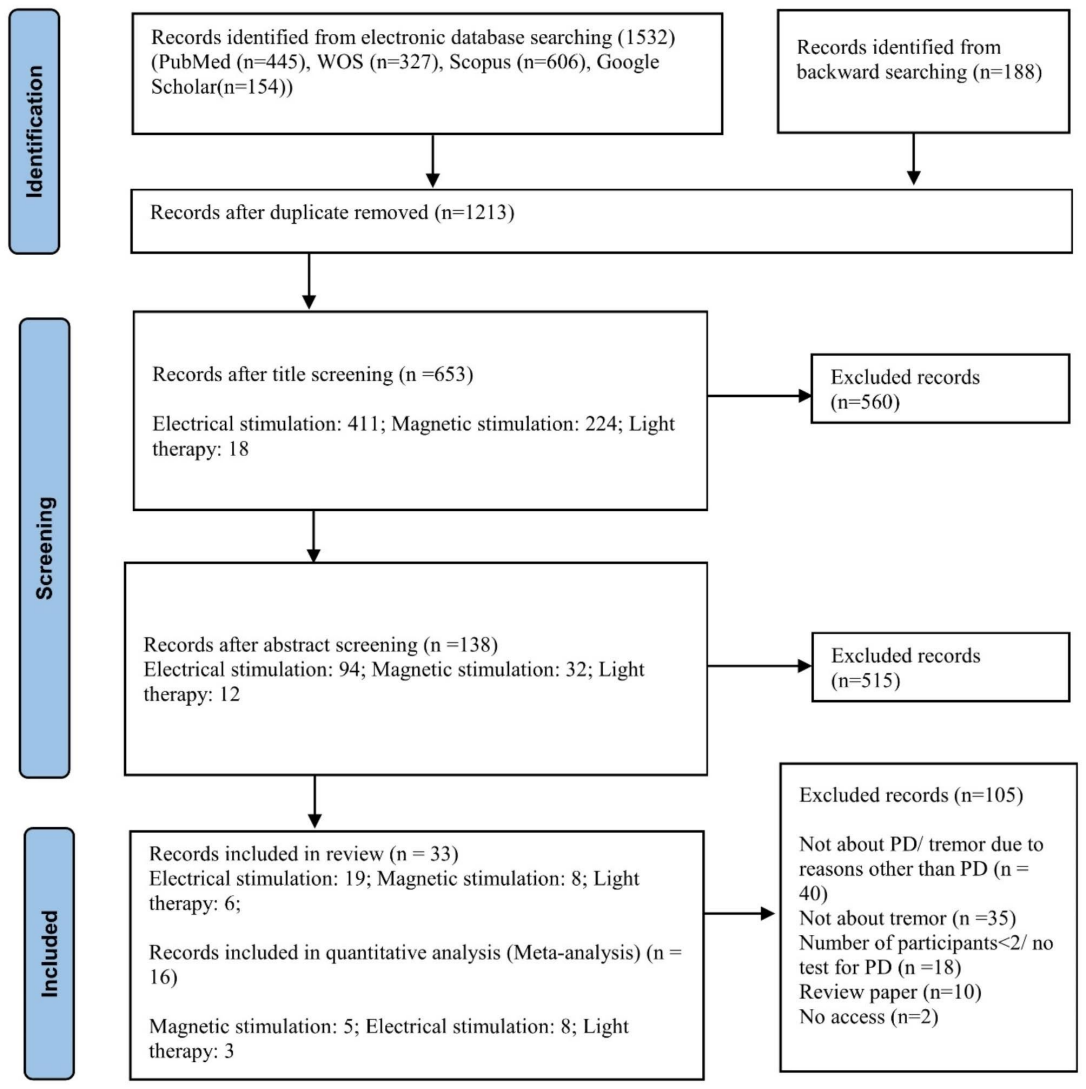
PRISMA flow diagram for searching procedure of the current study

Figure 1 showed the PRISMA flow diagram for this study. The included studies were shown in Table 1. Details of the studies can be found in Tables S1-S3 of the supporting materials.

The results of the meta-analyses were reported in the following sections. More information can be found in the supporting materials (sections B and C). All retrieved studies were published in English, except for Saavedra-Escalona et al. (2005) and Shi et al. (2020) which were published in other languages. Since recent studies have highlighted that for rare diseases inclusion of the results of studies with very low sample size may quantitatively improve the conclusion [32], relatively small sample-size studies (2 < n < 10) were also included in this study. However, as indicated in Table 1 in the main text, the majority of included studies (except Xu 201 and Javidian 1992) in the meta-analysis had relatively large sample size.

### Light therapy

For the effectiveness of light therapy on Parkinson’s disease tremors, the systematic search found 6 eligible studies with human samples (105 PD samples). Among the eligible studies, two of them [13, 15] showed a significant tremor reduction following light therapy; however, in [15] the effect was only observed for rest tremor. Light with different frequencies, including bright light [13], and the red to near-infrared [14, 33] range, showed a positive effect on tremor reduction, while according to other studies white fluorescent, polychromatic, and red light exposure were not effective in reducing tremors [11, 34]. The way in which light was applied varied between studies. Transmission of transcranial 670 and 850 nm light using a head-surrounding helmet [35], white fluorescent light applied to the head from a distance of 20 cm [13], and 940 nm near-infrared light applied to the posterior of the neck directed toward midbrain [15] were among strategies.

Considering all types of study designs, Bayesian meta-analysis obtained the pooled effect of SMD=-0.407(CrI= [-0.76, -0.066], 84 samples) for the effect of light therapy on tremor reduction in PD patients. In the case of including of only pre-post interventions with UPDRS III measure, SMD was − 0.44 (CrI= [-0.84,-0.03], 48 samples). The pooled effect indicated a positive impact of light therapy on tremor reduction. The estimated between-study heterogeneity was$\tau =0.19$ (95%CI= [0.01, 0.51]), which was relatively smaller than the initial best guess in the prior distribution of the model (i.e. 0.3). Other measures for assessing between-study heterogeneity showed negligible heterogeneity (I^2^ = 0.00%, H^2^ = 1, PI showed mostly the same sign as the pooled effect). For publication bias, Egger’s regression test, Begg’s rank test, and Thompson’s test (see Table 2) did not indicate the presence of funnel plot asymmetry or publication bias (P = 0.3, 0.056, and 0.46, respectively).

**Table 2:** Assessment of publication bias for different tremor reduction methods

Method	Egger’s test		Begg’s test		Thompson’s test	
	t	P	z	P	t	P
Light therapy	-1.194	0.3	-1.91	0.056	-0.81	0.46
Electrical stimulation	0.213	0.84	-0.08	0.94	0.21	0.84
Magnetic stimulation	0.86	0.43	0.61	0.54	1.30	0.25

### Magnetic stimulation

Our systematic search retrieved 8 studies with human samples regarding the effect of magnetic stimulation on PD tremors [12, 36–42]. The total PD sample size for these studies was n = 237. Studies mostly used transcranial magnetic stimulation for activating the cortex. Different measures using EMG or indices like UPDRS III or HY were used for evaluating the effect of magnetic stimulation on tremors. Five studies [12, 36, 38, 40, 42] reported a positive effect of TMS on tremor reduction, while two studies reported significant tremor reduction through the application of magnetic stimulation [36, 38]. Additionally, five studies provided enough information for calculating pooled effect size. Two studies used different stimulation parameters [38] or measures for tremor assessment [12]. Therefore, in total seven effect sizes were available for meta-analysis. Considering all types of study designs, the Bayesian meta-analysis obtained the pooled effect of SMD=-0.804(95%CrI= [-1.45; -0.08], 187 PD samples), indicating a significant effect of magnetic stimulation on PD tremor reduction.

Between-study heterogeneity for included studies was substantial ($\tau$=0.82, 95%CI= [0.41, 1.56]); therefore, in the first step, the studies were rechecked for possible outlier studies (using *find.outliers* function in *dmetar* R package). Subsequently a subgroup analysis was conducted according to the measure for assessing tremor (i.e. UPDRS III vs. other measures). The analysis showed that the study conducted by Malling et al. (2019) might contribute to the observed heterogeneity, However, its removal did not resolve between-study heterogeneity. According to the excitation protocol, three studies [12, 38, 43] used rTMS for stimulation of the motor cortex (five effect sizes). All of these studies used UPDRS III for evaluating tremors. The pooled effect for these studies was SMD= -0.65 (95%CrI=[-0.97, -0.27]), $\tau$ =0.00, I^2^ = 34.7%. Among these studies, two of them used high-frequency rTMS (10 Hz bilateral rTMS applied to M1 area using an H coil [42], and 20 Hz unilateral rTMS applied to M1 area using a figure of 8 coil [38]), while in [12, 38] a low-frequency rTMS excitation was tested for tremor reduction (1 Hz over motor cortex using figure of 8 coil). The subgroup analysis according to the frequency of rTMS obtained pooled effect of SMD=-0.81 (95%CrI=[-1.34, -0.2], $\tau$=0.26) for high-frequency rTMS and SMD=-0.22 (95%CrI=[-0.71, 0.32], $\tau$=0.23) for low-frequency rTMS. This result indicated that low-frequency rTMS may not be as effective as high-frequency intervention. Considering only pre-post study type, the pooled effect was SMD=-0.53 (95%CrI=[-0.93, -0.07], $\tau$=0.27). According to Table 2, none of the tests for publication bias found evidence for publication bias (p = 0.43, 0.54, 0.25, for Egger’s, Begg’s, and Thompson’s tests, respectively).

### Electrical stimulation

One dominant strategy for reducing PD tremors has been muscle/nerve stimulation using electrical pulses following the detection of tremors [44]. The systematic search found 19 eligible studies regarding the effectiveness of electrical stimulation on PD tremors with human samples (194 PD samples). Nine studies [17, 45–52] reported tremor suppression following electrical stimulation, while two other studies [53, 54] reported the worsening of tremor status after stimulation. In one study, both suppression and amplification of tremors were observed after electrical stimulation in different cases [50]. Since some recent systematic reviews have been performed on the effectiveness of electrical stimulation on PD tremor reduction, details for such strategies were not summarized in this study and readers were referred to Pascual‑Valdunciel et al. and Lora-Millan et al. for more detailed information [17, 55]. In brief, the EMG amplitude was recorded using surface or intramuscular electrodes, body movement was recorded by accelerometers, gyroscopes, motion sensors, or displacement sensors and measure such as the UPDRS tremor index were used for tremor detection. The amplitude and frequency of tremor, joint angle and UPDRS tremor index were the common measures for assessing the effectiveness of electrical stimulation on tremor. It should be noted that this study only considered non-invasive methodologies and studies like Arle et al. in which electrical stimulation was applied invasively were not considered [56].

According to Table 1, eight studies contained sufficient information for calculating the pooled effect (including 12 total effects). The Bayesian framework was used for the meta-analysis. The pooled SMD was − 0.36(95%CrI= [-0.67, -0.05]). Between-study heterogeneity was $\tau$=0.16(95%CI= [0.01, 0.52]) that indicated a small amount of heterogeneity between studies. A subgroup analysis according to the measure used for tremor assessment (i.e., tremor amplitude, frequency, or UPDRS scale) was also performed. The result of the subgroup analysis showed that when tremor was assessed based on amplitude (n = 3), SMD was − 0.63 (95%CrI= [-1.46; 0.16]), $\tau$=0.316, I^2^ = 11.3%, when the tremor frequency was considered (n = 5), SMD was 0.04(95%CrI= [-0.52;0.62]), $\tau$=0.0, I^2^ = 0.00%, and when UPDRS III (motor score) was used as a measure for tremor assessment (n = 4), pooled SMD was − 0.430 (95%CrI= [-0.94, 0.02]),$\tau$=0.27, I^2^ = 2.7%. It should be noted that studies with UPDRS III score were all pre-post studies. This result showed that the output measure for assessing tremor may be considered a confounding factor. According to Egger’s, Begg’s and Thompson’s tests, there was no evidence of publication bias (p = 0.84, 0.94, 0.84, respectively (see Table 2)).

### Between-study heterogeneity for different methods

Between-study heterogeneity of the included studies for meta-analysis was reported in Table 3. Four different measures (I^2^ statistic, H, PI, and $\varvec{\tau }$) were used for assessment.

**Table 3:** Assessment of between-study heterogeneity for different electromagnetic radiation tremor reduction methods. To compensate confounding factors, only studies with UPDRS III and pre-post design were considered

Method	I^2^%(95%CI)	H (95%CI)	PI	$\varvec{\tau }$ (range)
Light therapy	0.0 (0.0; 79.2)	1.00 (1.00;2.19)	(-1.01;0.11)	0.00 (0.00;0.96)
Electric stimulation	0.00 (0.0; 84.7)	1.00 (1.00;2.56)	(-1.52;0.52)	0.11 (0.00; 0.98)
Magnetic stimulation	43.0 (0.0; 79.1)	1.32 (1.00;2.19)	(-1.68;0.56)	0.29 (0.00; 1.22)

### Publication bias assessment and the quality of studies

The publication bias was assessed using Egger’s, Begg’s, and Thompson’s test, and the results were shown in Table 2. The quality of the studies was assessed using different checklists (refer to the supporting materials, section C). Among the 17 studies retrieved for electric stimulation, 10 studies had moderate to high quality, while the quality of seven studies was poor (see Tables S4-S5). For light therapy, among five studies, four of them were high-quality studies (Tables S5 and S6). For the magnetic stimulation strategy, three studies were of low quality, while other studies were of high or moderate quality (Tables S4-S7).

### Comparison between different methods for tremor suppression

**Fig. 2:**
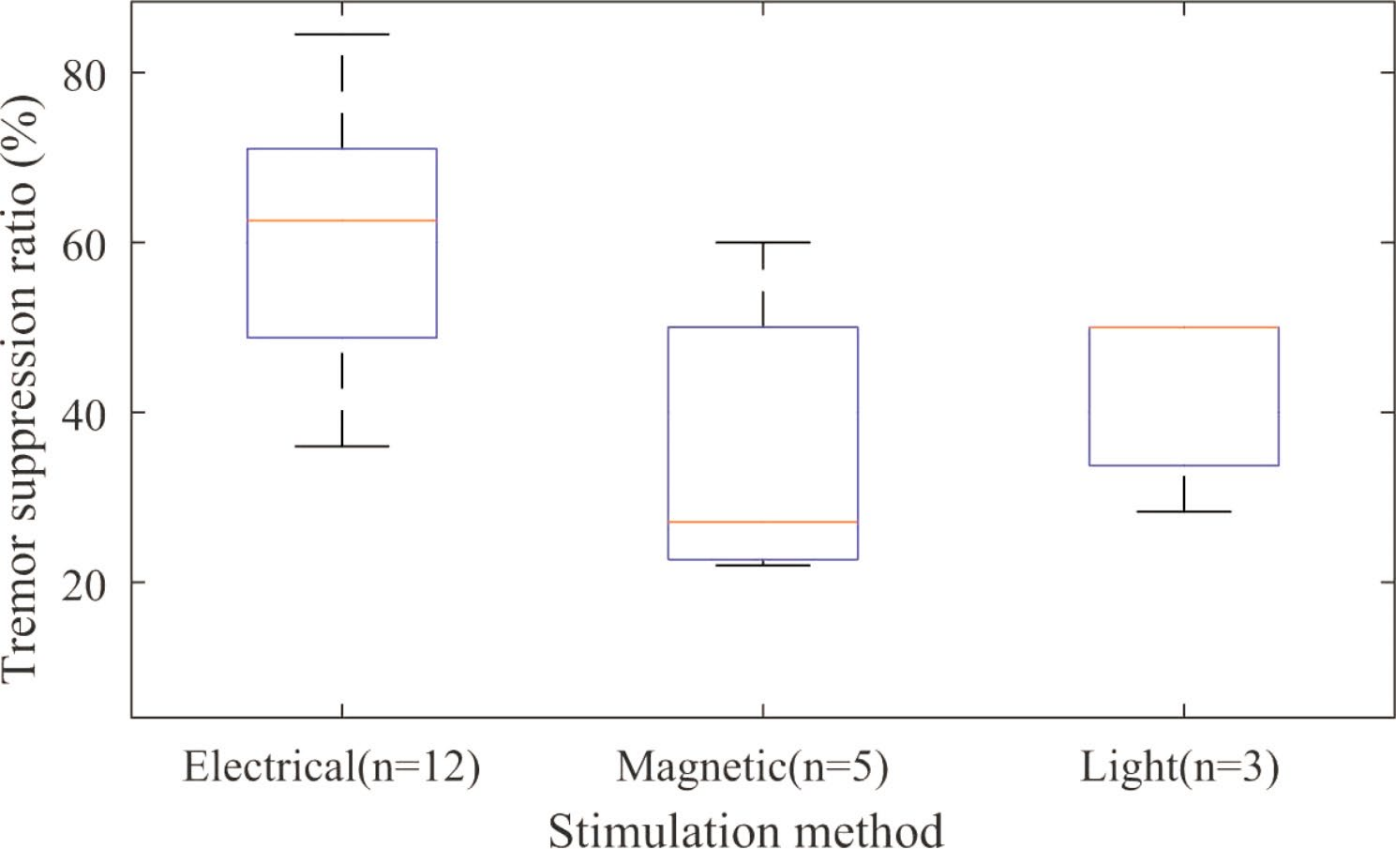
shows the box plots for the suppression ratio (%) of different methods for tremor suppression. Since the range of reported values for different methods was relatively broad, the median measure was used

Figure 2. **Comparison between suppression ratios of different methods for PD tremor suppression. In each box, the median percentage, maximum, minimum, first and third quartile were shown. The number of studies for each category was specified on the label.**

**Fig. 3:**
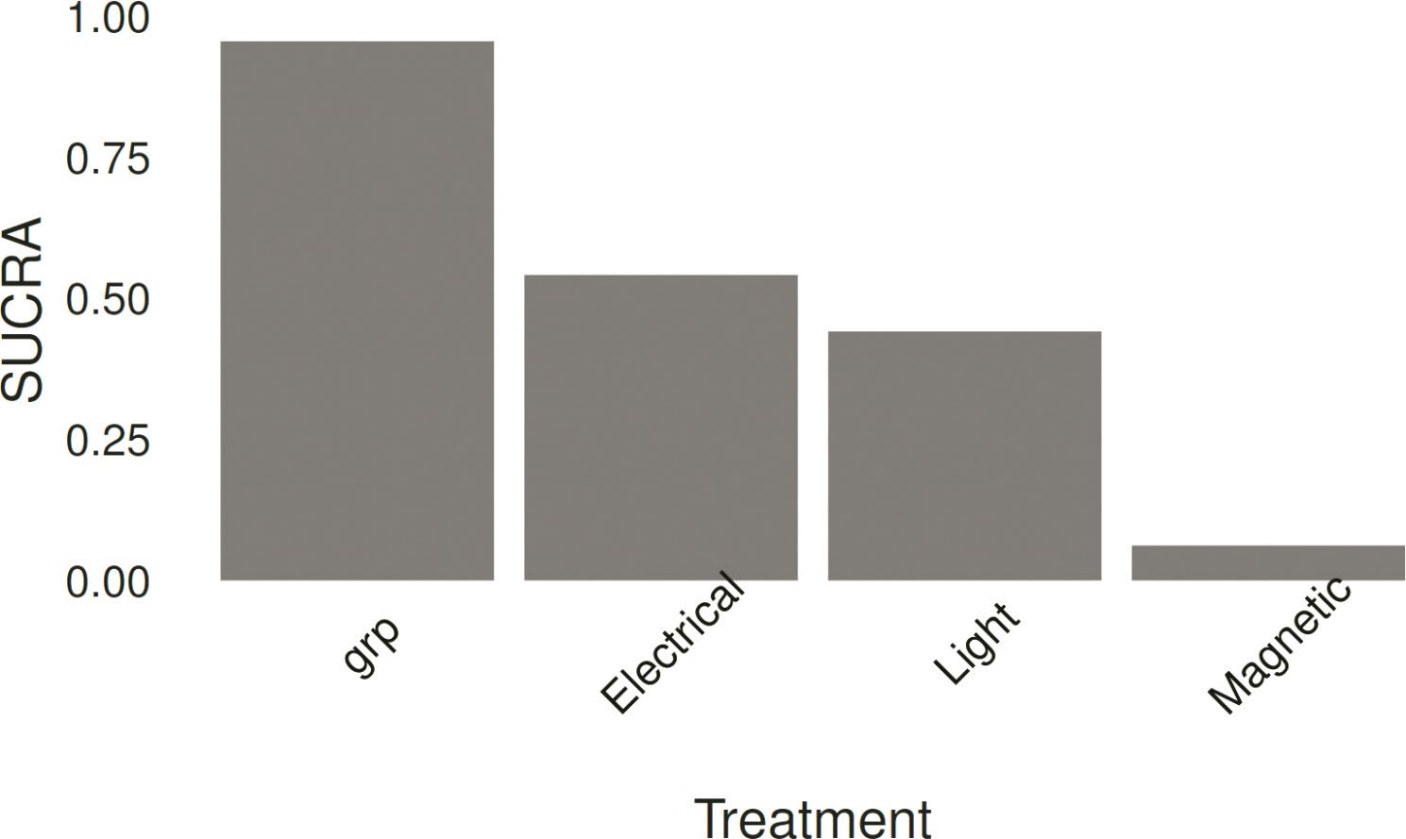
The SUCRA plot for ranking of different methods in tremor suppression

After checking for the convergence of the Bayesian network model, three electromagnetic-based intervention tremor suppression strategies were compared using the Bayesian network model. Using the *rank.probability* function in the *gemtc R package*, the probability of a treatment to be the best option was estimated. The Surface Under the Cumulative Ranking (SUCRA) score [57] was calculated for each method, and the result was shown in Fig. 3. SUCRA is a number between 0 and 100%, with a higher value (closer to 100%) indicating a higher likelihood of a therapy being ranked at the top [58]. In Fig. 3, grp indicated the pre-stimulation condition in which other methods were compared.

When applying an intervention for tremor suppression, it is interesting to know how the possible beneficial effects correlate with the patient’s disease severity and disease duration. In Table 4, the correlation between effect size/tremor suppression ratio and disease severity/duration was reported for each strategy. Pearson’s correlation was calculated, and the statistical significance was evaluated using a p-value.

**Table 4:** Correlation of effect size, suppression ratio, disease severity and disease duration. n indicates the number of studies for correlation analysis and dashed line indicated that no data was available for calculating the correlation

Strategy		Disease severity (UPDRS)	Disease duration (Year)
Light therapy	Effect size (SMD)	0.12 (p = 0.88), n = 4	
	Tremor suppression (%)	-0.36 (p = 0.76), n = 3	
	Tremor suppression (%)	-0.27 (p = 0.66), n = 5	0.02 (p = 0.96), n = 7
Magnetic stimulation	Effect size (SMD)	-0.98 (p = 0.003), n = 5	-0.33 (p = 0.53), n = 6
	Tremor suppression (%)		0.37 (p = 0.63), n = 4
Electrical stimulation	Effect size (SMD)	-0.92(p = 0.26), n = 3	-0.09 (p = 0.91), n = 4
	Tremor suppression (%)	0.40 (p = 0.43), n = 6	0.20 (p = 0.80), n = 4

## Discussion

### Light therapy

The result of the Bayesian meta-analysis revealed a positive effect of light therapy for tremor reduction in PD cases (SMD=-0.44 (CrI= [-0.84, -0.03]), no between-study heterogeneity and no publication bias). Light therapy may suppress melatonin [11] as an antioxidant against the pro-oxidant effects of L-dopa and dopamine. Furthermore, light therapy may influence PD through neuroprotective effects or by preventing oxidative stress inside the cells (see [59]). In light therapy, the weak penetration depth of the light prevents it from reaching deep brain areas which contain dopaminergic neurons, and in this regard, the impact of light therapy on PD symptoms may be restricted.

### Magnetic stimulation

According to the performed Bayesian meta-analysis, magnetic stimulation strategies were found to be effective for tremor suppression (SMD=-0.80 (95%CrI= [-1.45; -0.08])). According to the literature, such improvement may be attributed to the increased dopamine release following magnetic stimulation [60], the excitability of intracortical inhibitory circuitry [61], cortical excitability changes affecting synaptic plasticity [38], inhibition of test motor evoked potentials [62] and the change in circulation in brain regions are among suggested mechanisms for the effectiveness of magnetic stimulation on tremor suppression. Heterogeneity between studies regarding study design (excitation intensity, duration, frequency, or measure for tremor assessment) prevented us to investigate the exact effect of each parameter on the obtained results.

### Electrical stimulation

The pooled effect of electrical stimulation on tremor reduction was SMD=-0.36(95%CrI= [-0.67, -0.03]). Possible mechanisms for such an effect might be inhibition of the spinal stretch reflex through electrical nerve stimulation [63], modulation of tremor frequency by nerve stimulation [53], modulation of the peripheral reflex mechanism by electrical stimulation [46], and the generation of forces within the muscle, stimulating agonist-antagonist muscles and producing opposite forces to suppress handshaking [64].

### Comparison between different methods for tremor suppression

Considering the median value for the suppression ratio (Fig. 2), methods could be ordered as electrical stimulation, light therapy, and magnetic stimulation. This analysis highlighted the effectiveness of electrical stimulation. Furthermore, a comparison between different tremor reduction strategies using SUCRA measures (Fig. 3) from fitted Bayesian network obtained the same order. The included studies had different types of study designs. To compensate for the effect of study design and measure for tremor assessment, if only studies with a pre-post design and UPDRS III (motor section) measure were considered (since this type was the most prevalent among three strategies), the pooled effect size for electric, magnetic, and light stimulation were − 0.430 (95%CrI= [-0.94, 0.02]), -0.53 (95%CrI= [-0.93, -0.07]), and − 0.44 (CrI= [-0.84, -0.03], respectively. This adjusted comparison did not show better performance for electric stimulation compared to light or magnetic strategies. This result was obtained with low to moderate heterogeneity between studies (Table 3). According to Table 4, for light therapy, a positive correlation showed that the effect of the intervention was higher for severe cases, even though the correlation was weak (*r* < 0.5) and non-significant. While the strong negative correlation (*r* > 0.9) between electrical and magnetic stimulation showed that PD individuals with severe disease symptoms (higher UPDRS score) responded less effectively to the intervention for reducing tremor. This result was significant for magnetic stimulation (p < 0.05). According to the results of Table 4, for less severe cases, electrical and magnetic stimulation (*r* = 0.98, p = 0.66 and *r* = 0.92, p = 0.26, respectively) were found to be the most effective choices. The depth of penetration of electrical and magnetic stimulation is limited when applied superficially to the brain or peripheral regions. In tDCS experiments, the target areas are mainly cortical regions, while deep brain areas are not affected [65]. PD patients demonstrated a significantly greater reduction in cortical thickness than controls. Furthermore, several studies indicated that in more advanced stages of PD, cortical thickness was significantly degraded compared to early stages of the disease [66]. Structural degradation of the cortical region, which is the site of action for electrical and magnetic stimulation, might be the possible reason for the lower efficacy of electrical stimulation in the advanced stage of PD. For peripheral nerve stimulation, one possible mechanism for the effect of electromagnetic intervention on tremor reduction is by interrupting the tremor signal to the tremor source through the afferent fibers [17]. Diminished muscle afferent signaling and the progressive degeneration of brain structure during PD progression [67] may limit the potential of muscle and nerve electrical and magnetic stimulation for tremor reduction in more severe PD cases. Despite the above explanation, the correlation between the effectiveness of PD tremor reduction methods and the severity of disease should be carefully evaluated in future studies.

The correlation between effect size and disease duration indicated that for all strategies, by increasing disease duration the effect size of interventions was reduced.

## Conclusion

Non-pharmacological, non-surgical, and non-invasive methods, such as electrical stimulation, light therapy, and transcranial magnetic stimulation have been the center of attention for tremor reduction during the past decades. Comparison between such methodologies and investigating the causal relationship between the outcomes and confounding factors such as age and disease duration are lacking in the literature. This study was performed to add missing knowledge. According to obtained results of the current study, using electric, magnetic and light therapies were found to be effective in PD tremor suppression. Using suppression effectiveness and effect size level, tremor-suppressing methods can be arranged as electrical stimulation, light therapy, and magnetic stimulation therapy. Furthermore, the results showed that electrical and magnetic stimulation had better suppression effectiveness for the early stages of PD, while light therapy was a better choice for the late stage of the disease. It should be mentioned that due to the small size of included studies in each treatment category, the heterogeneity between studies due to different design, different measures for tremor assessment and more importantly small patient samples in the included studies the outcomes of this study should not be considered as a clinical guideline and more studies are required for checking the clinical significance, advantages, and disadvantages of each category.

### Electronic supplementary material

Below is the link to the electronic supplementary material.

Supplementary Material 1

## Data Availability

All data generated or analyzed during this study are included in this manuscript and the supplementary information file.
