# Supplementary material for: Electromagnetic radiation therapy for Parkinson’s disease tremor reduction- systematic reviews and Bayesian meta-analyses for comparing the effectiveness of electric, magnetic and light stimulation methods
Source: J Neuroeng Rehabil. 2023 Sep 26;20:129. doi: 10.1186/s12984-023-01255-z (PMC10521577; doi:10.1186/s12984-023-01255-z)
Supplement: Supplementary file 1 — Supplementary Material 1 [file 12984_2023_1255_MOESM1_ESM.docx]

**Section A. Detail information of included studies**

| Table S1. Summary of studies for the effect of Light therapy on tremor in human PD samples, Pre-Post: Pre-Post intervention; HY: Hoehn & Yahr scale; NR: Not reported; Int: Intervention; Con: Control. | | | | | | | | | | | | |
| --- | --- | --- | --- | --- | --- | --- | --- | --- | --- | --- | --- | --- |
| **Tremor reduction ratio (%)** | **Effect size (SMD (95% CI))** | **Main effects** | **Tremor measurement** | **Intervention description** | **PD severity (UPDRS or Hoen-Yahr scale)** | **Duration of symptoms** | **Age (mean ± STD)** | **Exposure tool/method** | **Number of males** | **Sample size^*^** | **Study type** | **Study (First Author, Year)** |
| 28.3% (according to UPDRS III, tremor subscore) | Within, action and postural tremors (UPDRS item 21): -0.303 (-0.96, 0.354),  Within, Rest tremors (UPDRS III): -0.65(-1.32, 0.02),  Within, overall tremors (UPDRS scores for parts I, II, III): -0.25 (-0.906, 0.406) | Overall tremors, action tremors and resting tremors after 2 weeks of treatment were reduced, however only the resting tremors showed significant improvement | UPDRS III | Near-infrared light (940nm, 6mw/cm^2^, 56.7Ma) exposed to posterior midline region of the neck daily. Photobiomodulation + H2 therapy for 2 weeks | HY: 2.35 | 6.15 ± 3.71 | 67.53 ± 8.83 | Hydrogen water and photobiomodulation | 12 | 18 | Pre-post | Hong, 2021 |
| 50% (number of patients reported improvement) | Cannot be determined | For two patients, light therapy was beneficial, for two others had no effect and for one case tremors were worsen. Another case had no tremors | NR | Photobiomodulation therapy using red to near-infrared light for 8 to 24 months. LEDs were installed inside a head cap or inserted to the nasal (670 and 810 nm LEDs twice daily for 10 -30 min) | Observation | 6.61 ± 5.24 | 66.67 ± 5.82 | Homemade intranasal LED device (660 nm); in-house built helmets, buckets with near-infrared light-emitting diode (LED)  devices of wavelengths across the red to near-infrared range | 6 | 6 | Case report | Hamilton, 2019 |
| NR | Within, red light: -0.27 (-1.411, 0.863)  Within, Polychromatic light: -1.148 (-2.368, 0.074) | No significant change for polychromatic light was found. While the light withdrawal deteriorated tremor | UPDRS III | Light (polychromatic, red, 3,000 lux, 0.8-1 m distance between diffuser and head) was administered by a ﬂuorescent tube directed to the sagittal plane of the head. All subjects were exposed to bright light for 4 months to 5 years before commencing the study | UPDRS:  Polychromatic light: 45.0 ± 28.8  Red light: 61.7 ± 30.4 | NR | 70.8 (Red light exposed); 66.9 (Polychromatic light exposed) | Light source containing ﬂuorescent tubes | 17 | 30 | Clinical trial | Willis, 2018^⸷+^ |
| 50% (number of patients reported improvement) | Cannot be determined | For two cases, light therapy reduced the tremor | NR | Transcranial red to near infrared light, 10- 30 minutes per day using a photobiomodulation helmet (670 nm LEDs after 3 months 850 nm LEDs) | NR | 3.83 ± 1.25 | 66.3 ± 7.57 | Inhouse photobiomodulation helmet, light emitting devices red to near infrared light | 3 | 3 | Case reports | Hamilton, 2018 |
| NR | Cannot be determined | Tremor was not affected by light therapy treatment | motor performance tests | White, fluorescent light (1000-1500 lux) for 1-1.5 hours before the sleep per day, for two to five weeks, assessment after at two weeks and five weeks | NR | 2-15 | 66 | light box consisting of ﬂuorescent tubes without ultra-violet emission | 8 | 12 | Clinical trial | Willis, 2007 |
| NR | Within, UPDRS III (tremor subscore): -0.28 (-0.937, 0.337), P<0.05  UPDRS III: -0.019(-0.67, 0.63), P>0.05 | Near significant tremor reduction after bright light therapy | UPDRS | Exposing white fluorescent by a light box every morning for 30 min for 15 days (10.000 lux), 1-hour after awakening, head-to-light distance of 20 cm, medication was preserved | HY: 2.7 ± 0.6 | 7.4 ± 4.3 | 63.6 ± 8 | Commercially white ﬂuorescent light boxes | 12 | 36 (Int: 18; Con:18) | Randomized controlled trial | Paus, 2007 |
| ^*^Sample size shows the number of samples who affected by Parkinson’s disease.  ^⸷^For effect size estimation, mean and standard deviation values were estimated using median and inter-quartile values according to [Hozo](https://bmcmedresmethodol.biomedcentral.com/articles/10.1186/1471-2288-5-13#auth-Stela_Pudar-Hozo) et al. (2005)([1](#_ENREF_1)).  ^+^ Independent samples for Polychromatic and red light exposed groups. | | | | | | | | | | | | |

| Table S2. Summary of studies for the effect of magnetic stimulation on tremor in human PD samples. Pre-Post: Pre-Post intervention; HY: Hoehn & Yahr scale; NR: Not reported; Int: Intervention; Con: Control | | | | | | | | | | | | |
| --- | --- | --- | --- | --- | --- | --- | --- | --- | --- | --- | --- | --- |
| **Tremor suppression ratio** | **Effect size (SMD (95% CI))** | **Main effects** | **Tremor measurement** | **Intervention description** | **PD severity ((UPDRS or Hoen-Yahr scale)**  **(UPDRS or Hoen-Yahr scale)** | **Duration of symptoms** | **Age (mean ± STD)** | **Exposure tool/method** | **Number of males** | **Sample size** | **Study type** | **Study (First Author, Year)** |
| Tremor score: 22.9 ± 31%  UPDRS III: 27.1 ± 16% | Within group: -1.00 (-1.48, -0.54) Between group: -0.72 (-1.27, -0.165), P=0.007 | Patients who received rTMS at M1-PFC and M1 showed signiﬁcantly  improvement in UPDRS part III total score (p = 0.007), tremor subscore (p = 0.011) | UPDRS III and UPDRS tremor subscore | 12 rTMS sessions, 4 weeks | 40.7 ± 10.6 | 6.7 ± 3.8 | 62 ± 9 | high-frequency rTMS and bilateral H5-coil | 41 | 59 (Int:39; Con: 20) | Randomized controlled trial | Spagnolo, 2021 |
| NR | Cannot be determined | rTMS combined with hyperbaric oxygen-acupuncture-rehabilitation therapy improves motor functions | UPDRS Ⅲ | 8-week treatment with western medicine in combination with rTMS and hyperbaric oxygen-acupuncture rehabilitation therapy | HY: 2.68 | 4.4 ± 1.5 | 62.4 ± 5.4 | rTMS combined with hyperbaric oxygen-acupuncture-rehabilitation therapy | 18 | 30 | Pre-post | Shi, 2020 |
| NR | UPDRS III: -0.361 (-0.91,0.187), P<0.05 | Tremor was improved after low frequency rTMS stimulation | UPDRS III | Bilateral stimulation at 1 Hz rTMS (each hemisphere two trains with interval of 30 s of 1000 stimuli) | 45.54 ± 20.01 | 4.60 ± 3.64 | 59.58 ± 11.28 | Low frequency rTMS (1Hz) | NR | 26 | Pre-post | Khedr, 2019 |
| NR | UPDRS III: -0.701 (-1.261, -0.141), P<0.05 | Tremor was improved after high frequency rTMS stimulation | UPDRS III | rTMS with 2000 pulses of 20 Hz rTMS (delivered in trains of 5 s with 25 s intervals) on motor cortex. | 46.46 ± 22.37 | 4.85±3.39 | 55.88±13.84 | High frequency rTMS (20 Hz) | NR | 26 | Pre-post | Khedr, 2019^*^ |
| 22% (tremor intensity)  32% (tremor peak power) | -2.61 (-0.83, 0.31), P=0.06 | T-PEMF treatment decreased inter-hand coherence in the PD group with unilateral. Also, tremor intensity decreased in both treatment groups in the resting | Tremor intensity | T-PEMF (30 min/day, 50 Hz, ±50 V, 3 ms squared pulses) | Int: 28±9; Con: 25±9 | Int: 6±5; Con: 3±2 | Int: 68±6  Con: 63±12 | daily transcranial PEMF treatment (T-PEMF) | 19 | 36 (Int: 24; Con:12) | Randomized controlled trial | Malling, 2019 |
| 46.7 ± 13.18% | Cannot be determined | M1 region is a critical contributor in resting and postural tremor in PD cases | test motor evoked potential (MEP) and long-interval intracortical inhibition | Determination of resting motor threshold (RMT), motor evoked potentials and long-interval intracortical inhibition duration | HY: 2.15 ± 0.53 | 3.05 ± 2.92 | 62.7 ± 11.41 | 30 single TMS pulses, intensity of 1mV randomly intermixed with 30 paired TMS pulses applied to M1, SMA and cerebellum | 6 | 10 | Case-Control | Lu, 2015 |
| NR | UPDRS III (tremor subscore): -0.03 (-0.91,0.85), P>0.06, UPDRS III (total): -0.179 (-1.06, 0.7), P>0.05 | No significant improvement was reported for tremor. | UPDRS III | rTMS (1800 stimuli at 1 Hz rate delivered to the motor cortex for four consecutive days | UPDRS: 46.8 ±11.6; HY: 3.3 ±0.7 | 15.6 ± 1.8 | 64.5 ± 3.0 | a standard Magstim ﬁgure-of-eight coil (0.2 T), rTMS session (1800 stimuli in total, duration 32 min) | 5 | 10 | Pre-post | Filipović, 2010 |
| 60% | HY: -1.135 (-1.698, -0.606), P<0.05 | partial or complete disappearance of tremor using the application of TMS | HY | Coils were placed on the patient’s scalp and the fields were applied for 6 minutes (2 minutes over each of the left and right temporal regions, frontal, and occipital regions, and over the vertex) | HY: 3.08 | 10.22 ± 5.69 | 65 (49-80) | TMS in the order of pico Tesla, magnetic amplitude: 1-7.5 pT, f: 8-13 Hz | 22 | 30 | Pre-post | Anninos, 2007 |
| NR | Cannot be determined | Magnetic stimulation significantly PD tremor period. | EMG and calculation of resetting index | motor cortex was stimulated by a TMS device. Stimulus intensity was 10% of muscle response threshold. 50 magnetic stimulations with 5-8 sec interval were applied. | NR | NR | 59 (range: 33-74) | The motor cortex was stimulated 19 turns of copper wire | NR | 10 | Case-Control | Britton, 1993 |

^*^ In this study, the sample size was divided to two sections and different analysis was done on each section. In this regard, this study was reported in two separated rows in the table.

| Table S3. Summary of studies for the effect of electrical stimulation on tremor in human PD samples. Pre-Post: Pre-Post intervention; HY: Hoehn & Yahr scale; NR: Not reported; Int: Intervention; Con: Control | | | | | | | | | | | | |
| --- | --- | --- | --- | --- | --- | --- | --- | --- | --- | --- | --- | --- |
| **Tremor suppression ratio** | **Effect size (SMD (95% CI))** | **Main effects** | **Tremor measurement** | **Intervention description** | **PD severity (UPDRS or Hoen-Yahr scale)**  **(UPDRS or Hoen-Yahr scale)** | **Duration of symptoms** | **Age (mean ± STD)** | **Exposure tool/method** | **Number of males** | **Sample size** | **Study type** | **Study (First Author, Year)** |
| NR | CD | bilateral tDCS and cathodal tDCS had the capability for suppressed Parkinsonian tremor | UPDRS Fahn-Tolosa-Marin Tremor Rating Scale, Purdue Pegboard Test , self-design Continuous Tremor Signal  Assessment | For bilateral setup, the right and left M1 hotspot were used for anode and cathode symmetrically. Direct current of 1.5 mA was delivered applied to the skull for 20 min. | 1.8 ±0.8 (HY) | 4.38 ±1.86 | 67.5 ± 4.9 | tDCS | 6 | 13 | RCT | Zhang, 2023 |
| NR | UPDRS III(tremor subscore): -0.558(-1.19,-0.074) | A significant reduction in tremor parameters during electric stimulation was observed ( reduction of UPDRS, and gyroscopic data. | UPDRS III (tremor score) | Starting stimulation parameter of 50 Hz and 150µs, increased intensity of pulses in 1mA until tremor reduction. Stimulation was continuous with a square-wave pulse and applied using a glove. | 2.53 ± 0.85 (HY) | 8.45 ± 3.26 | 63.40 ± 9.91 | Using two surface electrodes continuous stimulation with a square-wave pulse to the affected muscles | NR | 20 | Pre-Post | Phokaewvarangkul, 2021 |
| 36% | CD | Stimulation of median nerve can amplify and also reduce tremors depending on the phase modulation strategy. | Phase amplitude profile | Stimulation consisted of a five pulses burst at 7.7 ms interval. Each  phase was presented for 5 s and a 1 s interval. | HY I and II | NR | 65.5±8.3 | Phase-locked brief electrical pulses non-invasively to the median nerve of the most tremulous hand | 4 | 10 | Pre-Post | Arruda, 2021 |
| 50–71% of patients showed tremor reduction | Cannot be determined | Decrease in tremor amplitude was decreased significantly during and after stimulation in finger and after stimulation in hand and forearm. Main frequency was significantly reduced mainly during stimulation (53% to 68% mean tremor reduction) | Using tri-axial gyro-sensor, tremor amplitude and main frequency of each axis was calculated by power spectral density | Stimulation of wrist muscles: constant-current, monopolar-rectangular waveform, pulse width: 300 µs, f=100 Hz f | UPDRS III: 27.9±11.1; HY: 1.9±0.7 | 3.1±2.4 | 64.0±14.0 | Electrical stimulation of wrist muscles on sensory level | 7 | 14 | Pre-post | Heo, 2018 |
| 60 ± 30% | Cannot be determined | In average, the average optimal suppression level was 50%. The optimal stimulation setting was subjective. Low-current stimulation of afferent ﬁbers  can be considered as a promising approach for tremor suppression. | recordings of wrist movement, using an inertial measurement system | Stimulation of muscles using surface or intramuscular electrodes that delivered current-controlled, biphasic charge compensated pulses (100Hz, supra-threshold stimulation). | UPDRS: 17.8 ± 5.1 (range 9–21) | NR | 69.8 ± 7.0 | Surface or Intramuscular stimulation | 5 | 5 | Pre-post | Dideriksen, 2017 |
| 47.97 ± 25.77% | Cannot be determined | Electrical stimulation with intensities above 1.5 radiation threshold significantly reduced tremors. On average 47.97% tremor reduction was obtained. | Tremor amplitude and frequency using power spectral density calculation | Surface electrical stimulation using a train of biphasic current pulses (200 μs pulse width, 250 Hz pulse  frequency) at 6 stimulation sites | 18.5 ± 2.88 | 4.68 ± 2.76 | 63.75 ± 6.14 | Surface electrical stimulation of cutaneous afferents in the dorsal hand | 4 | 8 | Pre-post | Hao, 2017 |
| NR | UPDRS III (tremor): -0.509 (-1.537, 0.52), P=0.116 | Electrical muscle stimulation -based Tremor's glove effectively suppressed resting hand tremor in PD patients | UPDRS tremor section | An electrical muscle stimulation applied in hand  muscles via two self-adhesive electrodes | 31.13 ± 11.62 | 7.93 ± 4.01 | 63.40 ± 10.92 | Stimulation using a glove electrical muscle stimulation | 8 | 15 | Pre-Post | Jitkritsadakul, 2017^+^ |
| NR | Amplitude of joint angles (forearm pronation): -0.385 (-3.152, 2.436), P<0.05 | By electrical stimulation of cutaneous afferents, tremor was suppressed significantly in PD patients. | Motion capture system | Surface EMG data recorded from six muscles (biceps, triceps, flexor carpi ulnaris, extensor carpi radialis, flexor digitorum superficials, extensor digitorum), stimulation parameters: amplitude 1.0 mA, frequency 250 Hz, pulse width 200 µs | NR | NR | 69.5 ± 6.5 | Surface electrode on dorsal skin for stimulation | 1 | 2 | Pre-Post | Xu, 2016 |
| tremor amplitude: 49.57±38.89% | UPDRS tremor index: -0.88 (-1.584, -0.176), p<0.05  Peak amplitude: -0.558 (-1.243, 0.128), P<0.05  Tremor frequency: -0.206 (-0.88,0.468), P>0.05 | UPDRS tremor score reduced after electric stimulation. Furthermore, tremor amplitude and velocity were reduced (p<0.05) while tremor frequency remained unchanged (p>0.05). | UPDRS tremor index | 50 Hz electric stimulation exposed over the abductor pollicis brevis and interrosseus muscles for 10 seconds | UPDRS: 27.29 ± 7.56; HY: 2.67 ± 0.91 | 6.67 ± 2.86 | 65.29 ± 8.72 | Self-adhesive electrodes placed over the hand muscles most affected by tremor. Electrodes connected to stimulator | 18 | 34 | Pre-post | Jitkritsadakul, 2015 |
| Motor stimulation: 71 ± 5.35%  Sensory stimulation: 56.75 ± 6.55 % | Cannot be determined | An average tremor reduction (46%–81% and 35%–48% for motor and sensory stimulation) | Recording of EMG from the wrist ﬂexors and extensor muscles | Stimulation frequency was adjusted to 100 Hz, pulse width was adjusted to 300µs, and the current level was adjusted to a comfortable level for producing motor response | UPDRS: 20.5 ± 1.08 | NR | 69 ± 8.37 | Surface electrode for electrical stimulation of antagonist muscles in an out of phase manner, motor, and sensory stimulation | 3 | 4 | Pre-post | Dosen, 2015 |
| Tremor amplitude: 42.56 ± 24.91% | Cannot be determined | Neuroprosthesis provides systematic attenuation of tremor in PD | Kinematics, tremor power at wrist USING gyroscopes | Electrical stimulation of hand using a system with characteristics of: Pulse width: 250 or 300 µs, frequency: 30-40 Hz | 16.5 ± 16.5 | NR | 55.5 ± 11.5 | Electrodes placed over the ﬂexor carpi ulnaris and the biceps brachii | 2 | 2 | Pre-post | Gallego, 2013 |
| 63.6% | Amplitude of EMG at extensor digitorum*  -2.56 (-4.215, -0.879) | Instant suppression of tremor amplitude immediately after the start of electrical stimulation. | Surface EMG recorded  from the Biceps, triceps, FCU, ECR, FDS and ED muscles for 10-to 30 s | Surface muscle stimulation using biphasic current pulses,20 *s* duration, 100 *Hz* frequency, 200 µ*s* pulse width | NR | NR | NR | Bipolar non-woven surface electrodes placed on the  dorsal skin of the hand | NR | 10 | Pre-post | Hao, 2013 |
| 64.75 ± 15.3% | Cannot be determined | Adaptive out-of-phase stimulation attenuated the amplitude of tremor significantly | Inertial sensors (gyroscopes) | Controlled current sources that produce  biphasic compensated stimulation pulses, 1–50 pulses per second, pulse duration T = 50–500 µs, I = 0–50 mA | NR | NR | 59 ± 3.46 | Self-adhesive disposable stimulation electrodes placed on the forearm and upper arm, a programmable multichannel stimulator for asynchronous activation of several electrodes | 3 | 4 | Pre-post | Maneski, 2011 |
| 78.26 % | UPDRS: -0.199 (-0.806, 0.407), P<0.05 | Tremor reduction was observed in 78% of cases | Accelerometers | 10 minutes, 10 Hz, 250µs pulses | 49.04 | 8.29 ± 2.19 | 63.26 ± 12.67 | Electrical stimulation using stimulation electrodes | 12 | 23 | Pre-post | Saavedra-Escalona, 2005 |
| NR | Stimulation of opponens pollicis muscle: 0.273 (-1.12,1.66), P<0.05; forearm muscles: 0.386 (-1.13,1.78), P<0.05  Upper arm muscles: 0.356 (-1.04,1.75), P<0.05 | Resting Tremor frequency of PD increased significantly in the directly stimulated opponens pollicis muscle | tremor frequency using the mean burst-to-burst interval | Rhythmic electrical stimulation applied to the ipsilateral median nerve at the wrist | NR | 6.5 ± 4.2 | 65.25 ± 4.2 | Surface EMG from the opponens polli-cis muscle, the forearm extensors, and the forearm flexors using Ag/AgCl electrodes | 7 | 8 | Pre-post | Spiegel, 2002 |
| 84.5 ± 2.2 % | Cannot be determined | Using an electrical stimulation of muscles controlled by a digital controller, tremors were suppressed significantly | Using a three-dimensional accelerometer motion analysis system | Stimulating electrodes were placed on the skin in motor points of the wrist or ﬁnger ﬂexor and extensor muscles | NR | NR | 63–71 | Amplitude-modulated trains of electrical stimuli exposed to the extensor muscles and wrist ﬂexor | NR | 3 | Pre-post | Gillard, 1999 |
| 61.5 ± 6.18 % | Tremor frequency:  0.043 (-1.92,2.0), P>0.05 | PD tremor was attenuated to 62 % | Hand movement measuring using miniature displacement transducers |  | NR | NR | NR | self-adhesive electrodes placed at wrist or ipsilateral leg | NR | 4 | Pre-post | Javidan, 1992 |
| NR | Cannot be determined | Electrical stimulation of motor nerve inhibits or postpones the tremors for 183±16.3 ms in PD patients | EMG was recorded using surface electrodes | Electrical stimulation of radial nerve to excite extensor indicis and electrical stimulation of peroneal nerve at the knee.in tibialis anterior. (1 Hz, 1ms square waves) | NR | 7.5 ± 5.87 | 59.4 ± 8.30 | Electrical stimulus by a square-wave impulse of 1ms/1Hz stimulation | 6 | 10 | Pre-post | Bathien, 1980 |
| NR | Cannot be determined | After shock, no change in tremor amplitude, while tremor frequency was changed. Furthermore, the interval of the post-stimulation tremor bursts was reduced when compared with control values | EMG recording from hand muscles | Electrical stimulation characteristics: 500 µs, single shock stimulation | NR | NR | NR | Random single 0-5ms square-wave applied to the ipsilateral or contralateral olnar and median nerve | NR | 5 | Pre-post | Mones, 1969 |
| ^*^For this study effect size can be calculated for different muscles. Only the best result was reported to avoid dependent effect sizes.  ^**^For this study, trmor peak amplitude, frequency and RMS angle and velocity were reported for distinct directions (i.e. X, Y and Z). However to be consistent with other studies, these effects was not used for calculating the pooled effect size. | | | | | | | | | | | | |

**Section B. Forest plots for performed meta-analysis**

**
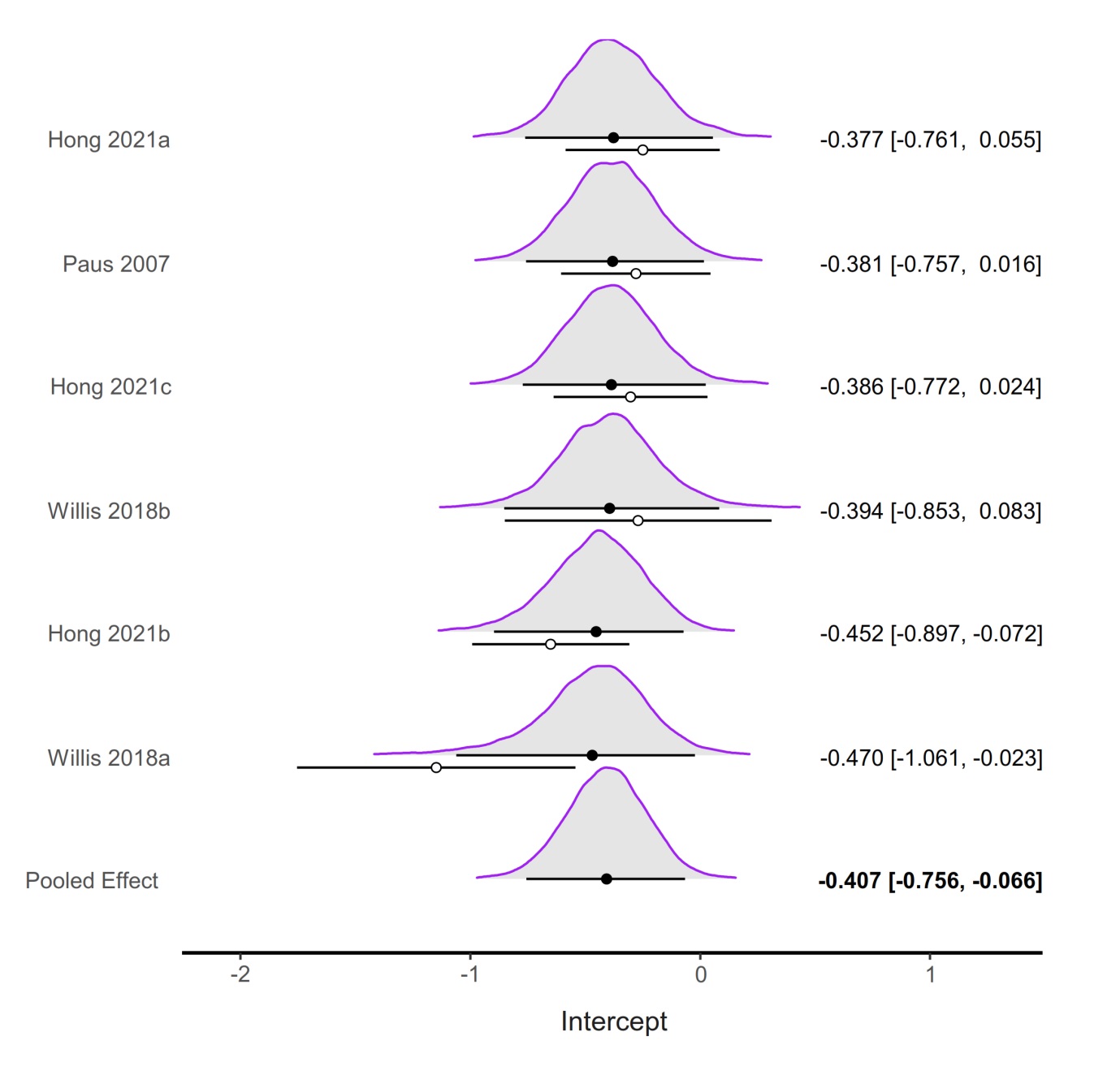
**

Fig S1. Forest plot for the effectiveness of light therapy on PD tremor. Letters in the study column indicated different effects obtained by different measure or independent groups. The empty dots indicated the real effect size of the study, while filled dots indicated the estimated effect size according to the fitted Bayes model. Each horizontal line indicated the corresponding confidence or credible interval.


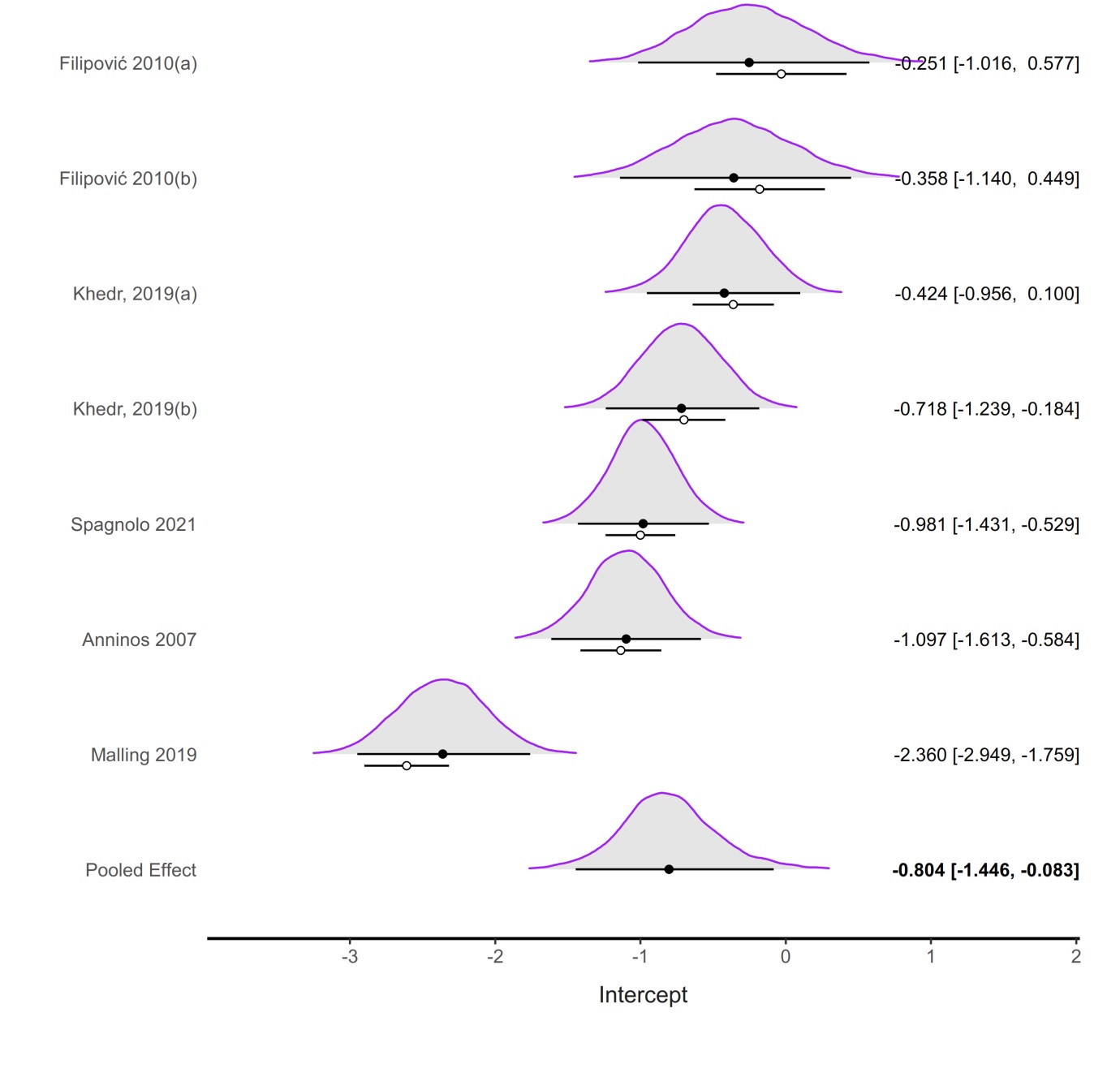
Fig S2. Forest plot for the effectiveness of magnetic stimulation on PD tremor. The distribution profiles were obtained through the Bayesian model. The empty dots indicated the real effect size of the study, while filled dots indicated the estimated effect size according to the fitted Bayes model. Each horizontal line indicated the corresponding confidence or credible interval. Letters in the study column indicated different effects obtained by different measure or independent groups.


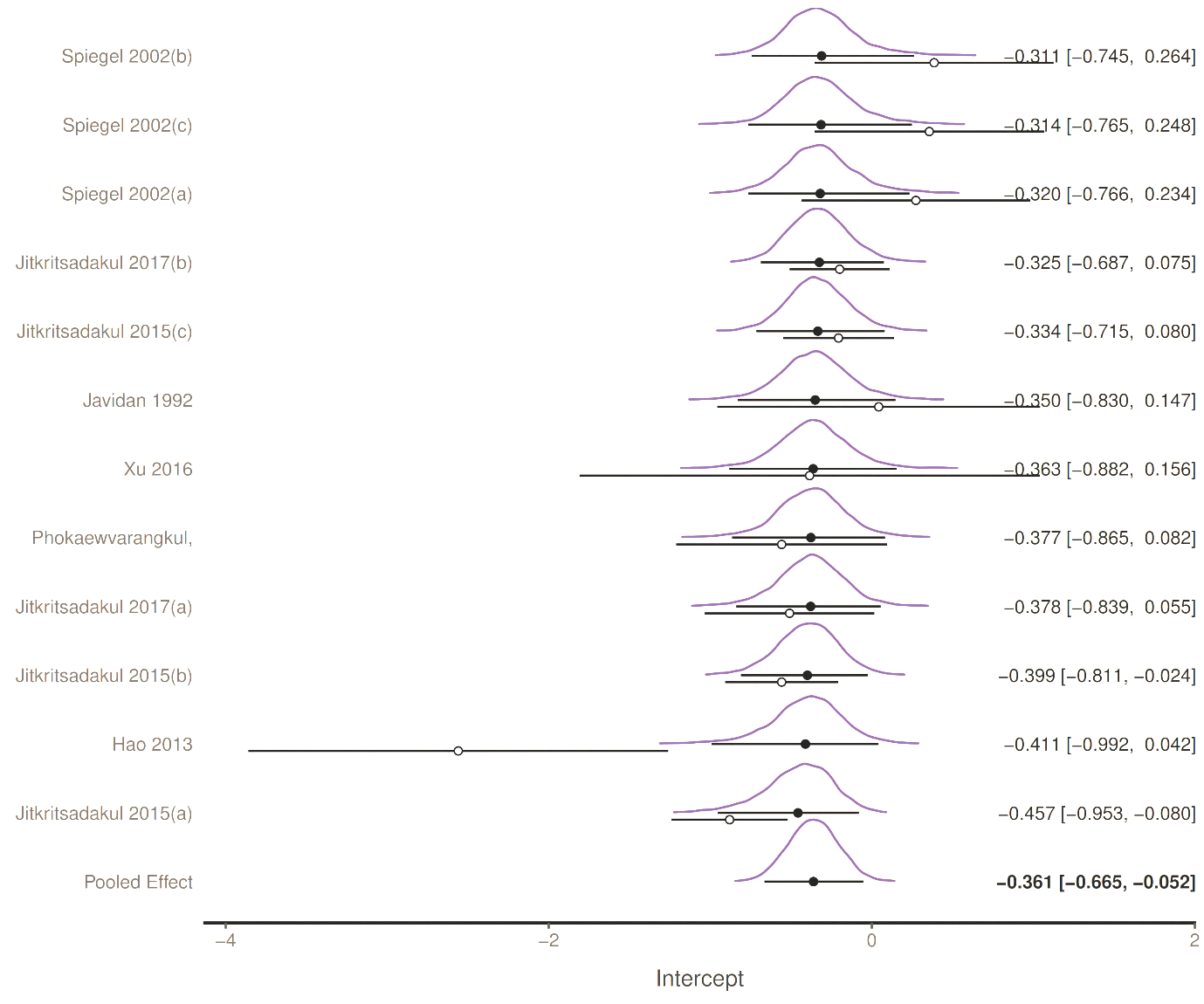


Fig S3. Forest plot for the effectiveness of electrical stimulation on PD tremor. The distribution profiles were obtained through the Bayesian model. The empty dots indicated the real effect size of the study, while filled dots indicated the estimated effect size according to the fitted Bayes model. Each horizontal line indicated the corresponding confidence or credible interval. Letters in the study column indicated different effects obtained by different measure or independent groups.

**Section C. Funnel plots for performed meta-analysis**


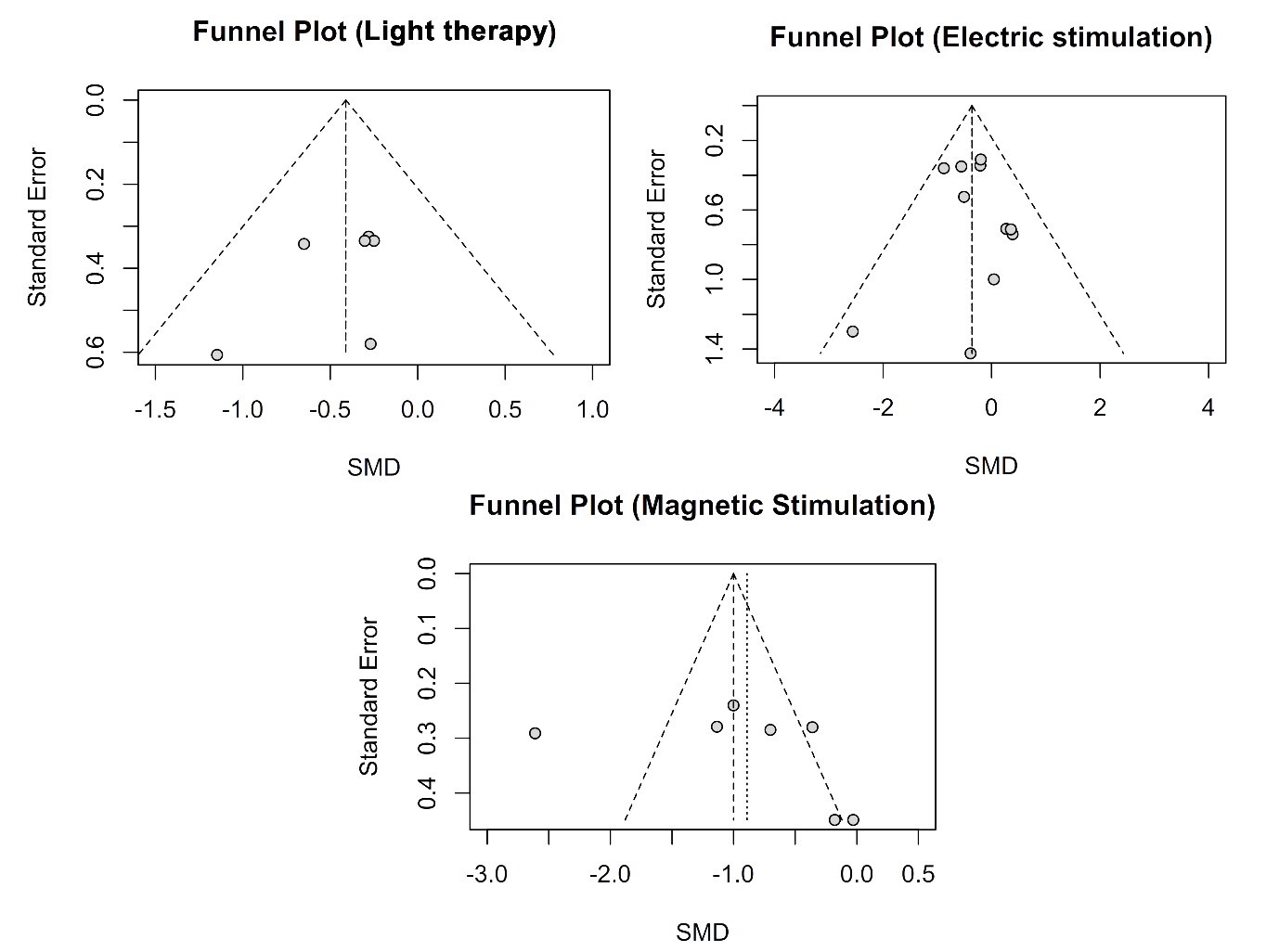


Fig S4. Funnel plot representation for assessing publication bias of different non-invasive, non-pharmacological tremor reduction methodologies.

**Section D: Quality assessment checklists**

Table S4. Quality assessment for Pre-Post studies with no control group ([2](#_ENREF_2)), (Y: Yes; N: No; Other **(**CD: cannot determine**,** NR: not reported**,** NA: not applicable**)**. Score≥6 (quality: Fair), Score≤6 (quality: Poor).

| Study | Category | 1 | 2 | 3 | 4 | 5 | 6 | 7 | 8 | 9 | 10 | 11 | 12 | Total score |
| --- | --- | --- | --- | --- | --- | --- | --- | --- | --- | --- | --- | --- | --- | --- |
| Phokaewvarangkul, 2021 | Electrical stimulation | Y | Y | N | Y | N | Y | Y | N | Y | Y | N | NR | 7 (Fair) |
| Arruda, 2021 | Electrical stimulation | Y | Y | N | N | N | Y | Y | NR | Y | Y | N | NR | 6(Fair) |
| Arruda, 2021 | Light therapy | Y | Y | N | N | N | Y | Y | NR | Y | Y | N | NR | 6 (Fair) |
| Anninos, 2007 | Magnetic stimulation | Y | Y | N | Y | N | Y | Y | NR | Y | Y | N | NR | 7 (Fair) |
| Filipović, 2010 | Magnetic stimulation | Y | N | N | Y | N | Y | Y | NR | Y | Y | N | NR | 6 (Fair) |
| Khedr, 2019 | Magnetic stimulation | Y | Y | N | Y | N | Y | Y | NR | Y | Y | Y | NR | 8 (Fair) |
| Shi, 2020 | Magnetic stimulation | Y | Y | N | N | N | Y | Y | CD | Y | N | N | NR | 5 (Poor) |
| Heo, 2018 | Electrical stimulation | Y | N | N | Y | N | Y | Y | NR | Y | Y | N | N | 6 (Fair) |
| Dideriksen, 2017 | Electrical stimulation | Y | N | N | Y | N | Y | Y | NR | Y | N | N | N | 5 (Poor) |
| Hao, 2017 | Electrical stimulation | Y | N | N | Y | N | Y | Y | NR | Y | Y | N | Y | 7 (Fair) |
| Xu, 2016 | Electrical stimulation | Y | N | N | Y | N | Y | Y | N | Y | Y | N | N | 6 (Fair) |
| Jitkritsadakul, 2015 | Electrical stimulation | Y | Y | N | Y | N | Y | Y | N | Y | Y | N | N | 7 (Fair) |
| Dosen, 2015 | Electrical stimulation | Y | N | N | Y | N | Y | Y | N | Y | Y | N | N | 6 (Fair) |
| Gallego, 2013 | Electrical stimulation | Y | N | N | Y | N | Y | Y | N | Y | N | N | N | 5 (Poor) |
| Hao, 2013 | Electrical stimulation | Y | N | N | Y | N | Y | Y | N | Y | N | N | N | 5 (Poor) |
| Maneski, 2011 | Electrical stimulation | Y | N | N | Y | N | Y | Y | N | Y | N | N | N | 5 (Poor) |
| Saavedra-Escalona, 2005 | Electrical stimulation | Y | Y | N | N | N | Y | Y | N | Y | N | N | N | 5 (Poor) |
| Spiegel, 2002 | Electrical stimulation | Y | Y | N | Y | N | Y | N | N | Y | Y | N | N | 6 (Fair) |
| Gillard, 1999 | Electrical stimulation | Y | N | N | Y | N | Y | N | NR | Y | N | N | N | 4 (Poor) |
| Javidan, 1992 | Electrical stimulation | Y | Y | N | Y | N | Y | Y | NR | Y | Y | N | N | 7 (Fair) |
| Bathien, 1980 | Electrical stimulation | Y | N | N | Y | N | Y | Y | N | Y | N | N | N | 5 (Poor) |
| Mones, 1969 | Electrical stimulation | Y | N | N | Y | N | Y | Y | NR | Y | N | N | N | 5 (Poor) |
| 1. Was the study question or objective clearly stated? 2. Were eligibility/selection criteria for the study population prespecified and clearly described? 3. Were the participants in the study representative of those who would be eligible for the test/service/intervention in the general or clinical population of interest? 4. Were all eligible participants that met the prespecified entry criteria enrolled? 5. Was the sample size sufficiently large to provide confidence in the findings? 6. Was the test/service/intervention clearly described and delivered consistently across the study population? 7. Were the outcome measures prespecified, clearly defined, valid, reliable, and assessed consistently across all study participants? 8. Were the people assessing the outcomes blinded to the participants' exposures/interventions? 9. Was the loss to follow-up after baseline 20% or less? Were those lost to follow-up accounted for in the analysis? 10. Did the statistical methods examine changes in outcome measures from before to after the intervention? Were statistical tests done that provided p values for the pre-to-post changes? 11. Were outcome measures of interest taken multiple times before the intervention and multiple times after the intervention (i.e., did they use an interrupted time-series design)? 12. If the intervention was conducted at a group level (e.g., a whole hospital, a community, etc.) did the statistical analysis take into account the use of individual-level data to determine effects at the group level? | | | | | | | | | | | | | | |

Table S5. JADAD score ([3](#_ENREF_3)) for quality assessment for randomized controlled trials (RCT), Randomized trials (RT) and Clinical trials (Score≥3 (quality high), Scores<3 (quality low)).

| Study | Category | Randomization | Blinding | Withdrawals | Total score (quality) |
| --- | --- | --- | --- | --- | --- |
| Paus, 2007 | Light therapy | 0 | 0 | 0 | 0 (low) |
| Willis, 2018 | Light therapy | 2 | 2 | 0 | 4 (High) |
| Malling, 2019 | Magnetic stimulation | 2 | 2 | 0 | 4 (High) |
| Spagnolo,2021 | Magnetic stimulation | 2 | 2 | 0 | 4 (High) |
| Jitkritsadakul, 2017 | Electrical stimulation | 2 | 2 | 0 | 4 (High) |
| 1. Was the study described as randomized (this includes the use of words such as randomly, random, and randomization)? (1 point if randomization is mentioned, 1 additional point if the method of randomization is appropriate, deduct 1 point if the method of randomization is inappropriate) 2. Was the study described as double blind? (1 point if blinding is mentioned, 1 additional point if the method of blinding is appropriate, deduct 1 point if the method of blinding is inappropriate). 3. Was there a description of withdrawals and dropouts? (1 point if the number and the reason for withdrawal in each group are stated) | | | | | |

Table S6. NIH quality assessment tool ([2](#_ENREF_2)) for case series/case reports (SCORE≥6 quality good, score<6 quality poor). Y: Yes; N: No; Other **(**CD: cannot determine**,** NR: not reported**,** NA: not applicable**)**.

| Study | Category | 1 | 2 | 3 | 4 | 5 | 6 | 7 | 8 | 9 | Total (Quality) |  |
| --- | --- | --- | --- | --- | --- | --- | --- | --- | --- | --- | --- | --- |
| Willis, 2007 | Light therapy | Y | Y | CD | CD | Y | Y | Y | N | Y | 6 (High) |  |
| Hamilton, 2018 | Light therapy | Y | Y | CD | N | Y | N | Y | Y | Y | 6 (High) |  |
| Hamilton, 2019 | Light therapy | Y | Y | CD | N | Y | Y | Y | Y | Y | 7 (High) |  |
| 1. Was the study question or objective clearly stated? 2. Was the study population clearly and fully described, including a case definition? 3. Were the cases consecutive? 4. Were the subjects comparable? 5. Was the intervention clearly described? 6. Were the outcome measures clearly defined, valid, reliable, and implemented consistently across all study participants? 7. Was the length of follow-up adequate? 8. Were the statistical methods well-described? 9. Were the results well-described? | | | | | | | | | | | | |

Table S7. Newcastle-Ottawa quality assessment tool ([4](#_ENREF_4)) for case-control and pilot studies (score≥7, high quality, score<7, low quality).

| Study | Category | 1 | 2 | 3 | 4 | 5 | 6 | 7 | 8 | Total score |
| --- | --- | --- | --- | --- | --- | --- | --- | --- | --- | --- |
| Britton, 1993 | Magnetic stimulation | 1 | 0 | 1 | 0 | 0 | 1 | 1 | 1 | 5 (Low) |
| Lu, 2015 | Magnetic stimulation | 1 | 0 | 1 | 1 | 0 | 2 | 1 | 0 | 6 (Low) |
| 1. Is the Case Definition Adequate? 2. Representativeness of the Cases 3. Selection of Controls 4. Definition of Controls 5. Comparability of Cases and Controls on the Basis of the Design or Analysis (two stars) 6. Ascertainment of Exposure (two stars) 7. Same method of ascertainment for cases and controls 8. Non-Response Rate | | | | | | | | | | |

References

1. Hozo SP, Djulbegovic B, Hozo I. Estimating the mean and variance from the median, range, and the size of a sample. BMC Medical Research Methodology. 2005;5(1):13.

2. Ma L-L, Wang Y-Y, Yang Z-H, Huang D, Weng H, Zeng X-TJMMR. Methodological quality (risk of bias) assessment tools for primary and secondary medical studies: what are they and which is better? Mil Med Res. 2020;7(1):1-11.

3. Jadad AR, Moore RA, Carroll D, Jenkinson C, Reynolds DJM, Gavaghan DJ, et al. Assessing the quality of reports of randomized clinical trials: is blinding necessary? Controlled clinical trials. 1996;17(1):1-12.

4. Wells GA, Shea B, O’Connell D, Peterson J, Welch V, Losos M, et al. The Newcastle-Ottawa Scale (NOS) for assessing the quality of nonrandomised studies in meta-analyses. Oxford; 2000.
